# Genotyping of canine MHC gene DLA‐88 by next‐generation sequencing reveals high frequencies of new allele discovery and gene duplication

**DOI:** 10.1111/tan.14752

**Published:** 2022-08-09

**Authors:** Chul‐Woo Pyo, Michael A. Harkey, Beverly Torok‐Storb, Rainer Storb, Ruihan Wang, Alexander S. Thomas, Wyatt C. Nelson, Daniel E. Geraghty

**Affiliations:** ^1^ Division of Clinical Research Fred Hutchinson Cancer Center Seattle Washington USA; ^2^ Scisco Genetics Inc. Seattle Washington USA; ^3^ Department of Medicine University of Washington Seattle Washington USA

**Keywords:** allele, canine, class I, DLA‐88, dog leukocyte antigen, major histocompatibility complex

## Abstract

Dogs have served as one of the most reliable preclinical models for a variety of diseases and treatments, including stem/progenitor cell transplantation. At the genetic epicenter of dog transplantation models, polymorphic major histocompatibility complex (MHC) genes are most impactful on transplantation success. Among the canine class I and class II genes, DLA‐88 has been best studied in transplantation matching and outcomes, with 129 DLA‐88 alleles identified. In this study we developed and tested a next generation (NGS) sequencing protocol for rapid identification of DLA‐88 genotypes in dogs and compared the workflow and data generated with an established DLA‐88 Sanger sequencing protocol that has been in common prior use for clinical studies. By testing the NGS protocol on a random population of 382 dogs, it was possible to demonstrate superior efficacy based on laboratory execution and overall cost. In addition, NGS proved far more effective at discovering new alleles and detecting multiple alleles associated with gene duplication. A total of 51 new DLA‐88 alleles are reported here. This rate of new allele discovery indicates that a large pool of yet un‐discovered DLA‐88 alleles exists in the domestic dog population. In addition, more than 46% of dogs carried three or more copies of DLA‐88, further emphasizing the need for more sensitive and cost‐effective DLA typing methodology for the dog clinical model.

**Abbreviations**
DLA: =; dog leukocyte antigen
FHCC: =; fred hutchinson cancer center
HLA: =; HLA
HSCT: =; hematopoietic stem cell transplantation
HVR: =; hypervariable region
IPD‐MHC: =; immuno‐polymorphism major histocompatibility complex database
MHC: =; major histocompatibility complex
NGS: =; next generation sequencing
PCR: =; polymerase chain reaction (Part of EMBL‐EBI)
EMBL‐EBI: =; European Molecular Biology Laboratory European Bioinformatics Institute

## 1 INTRODUCTION

The HLA genes in humans and HLA homologs in other mammals play an essential role in immune defense against infection and abnormal cells. They are clustered in the major histocompatibility complex (MHC) and encode transmembrane proteins with a peptide‐binding domain oriented on the exterior surface of the cell. Their primary function is to present an inventory of the antigens from in and around cells to the immune system.^1^ Many of these genes are highly polymorphic, with 100s to 1000s of known alleles in the human population.^2^, ^3^ Similar complexity likely exists in other mammals, but most have not yet been deeply analyzed in that regard. The polymorphism of these molecules concentrates in the antigen‐binding pocket, creating a diverse antigen recognition repertoire and allowing for the detection of a vast array of foreign antigens proportional to the level of polymorphism within a population. As a result, individuals vary in their immunity to pathogens, their predisposition to inflammatory disorders, and their compatibility in cell, organ, or tissue transplantation.^4^ The HLA genes also play a role in regulating the composition of the gut microbiome,^1^, ^5^, ^6^, ^7^, ^8^ which, in turn, plays important roles in a wide variety of health issues.^9^

Genotyping of HLA class I and II genes is essential for successful hematopoietic cell, tissue, and organ transplantation. These genes have also had longstanding status as markers for disease resistance, autoimmune disorders and, more recently, dysbiosis of the gut microbiome.^1^, ^5^, ^6^, ^7^, ^8^, ^9^ Due to the complex polymorphism of these genes, for dogs, which do not have an established catalog of known allele types as in the human system, genotyping has required extensive sequencing often including cloning, resulting in an expensive and time‐consuming process. Recent advances in next‐generation sequencing (NGS) have yielded much faster and less expensive approaches to HLA typing.^10^, ^11^ In addition to the clinical benefits, this approach has opened the door for large‐scale research programs in testing the associations between HLA polymorphism and a wide range of associated diseases.^12^, ^13^, ^14^, ^15^

Dogs provide an informative pre‐clinical model for transplantation of bone marrow stem cells, tissues, and organs.^16^, ^17^, ^18^, ^19^, ^20^ As with HLA in humans, genotyping of dog leukocyte antigen (DLA) genes is essential for matching donors to recipients for a successful graft,^21^, ^22^ and has become increasingly important in canine models of inflammatory disorders.^23^ However, the costs in time and money for DLA typing using conventional methods has limited the scope of research in these areas. The most polymorphic of the class I DLA genes and the most informative of successful tissue/cell engraftment is DLA‐88.^24^ Like class I HLA molecules in humans, the DLA‐88‐encoded protein binds and presents a wide variety of self and viral antigens.^25^, ^26^, ^27^

Here we describe an NGS approach to DLA‐88 genotyping. The approach was designed based on an amplicon‐based system for HLA typing^10^ that mirrors both the laboratory data acquisition steps and the computational analytical framework developed to determine genotypes using high‐throughput technology. Using this design, a panel of 382 dogs was examined to compare the NGS method to an established Sanger‐based DLA typing approach, comparing both rates of allele drop off and efficacy in the detection of novel alleles. Other metrics for comparison included the execution time, cost, and scalability to large sample numbers.

## 2 MATERIALS AND METHODS

### 2.1 DNA specimens

Blood samples were obtained from 286 dogs of random breeds from Phoenix Laboratories, Edmonds, WA, as discarded excess diagnostic samples. Samples were identified to us by a unique sample number. The identity of the dog, or the dog owner, was not provided. Genomic DNA was isolated from whole blood using either Puregene DNA purification kit (Gentra Systems, Minneapolis, MN) or QIAamp DNA blood mini kit (Qiagen, Valencia, CA). This group of dogs included members of 86 distinct breeds; however, it was not possible to accurately assign breed designations to individual animals in this group. For reporting, these samples were treated as a random group of dogs without reference to breed. To supplement the study to include samples where breed designation was assigned, additional DNA samples were obtained from the Cell and Molecular Services and Analysis Lab within the Cooperative Center of Excellence in Hematology (CCEH) at the Fred Hutchinson Cancer Center (FHCC) that were accurately assigned to 48 Great Danes and 48 German Shepherds.

### 2.2 Next generation sequencing

**FIGURE 1:**
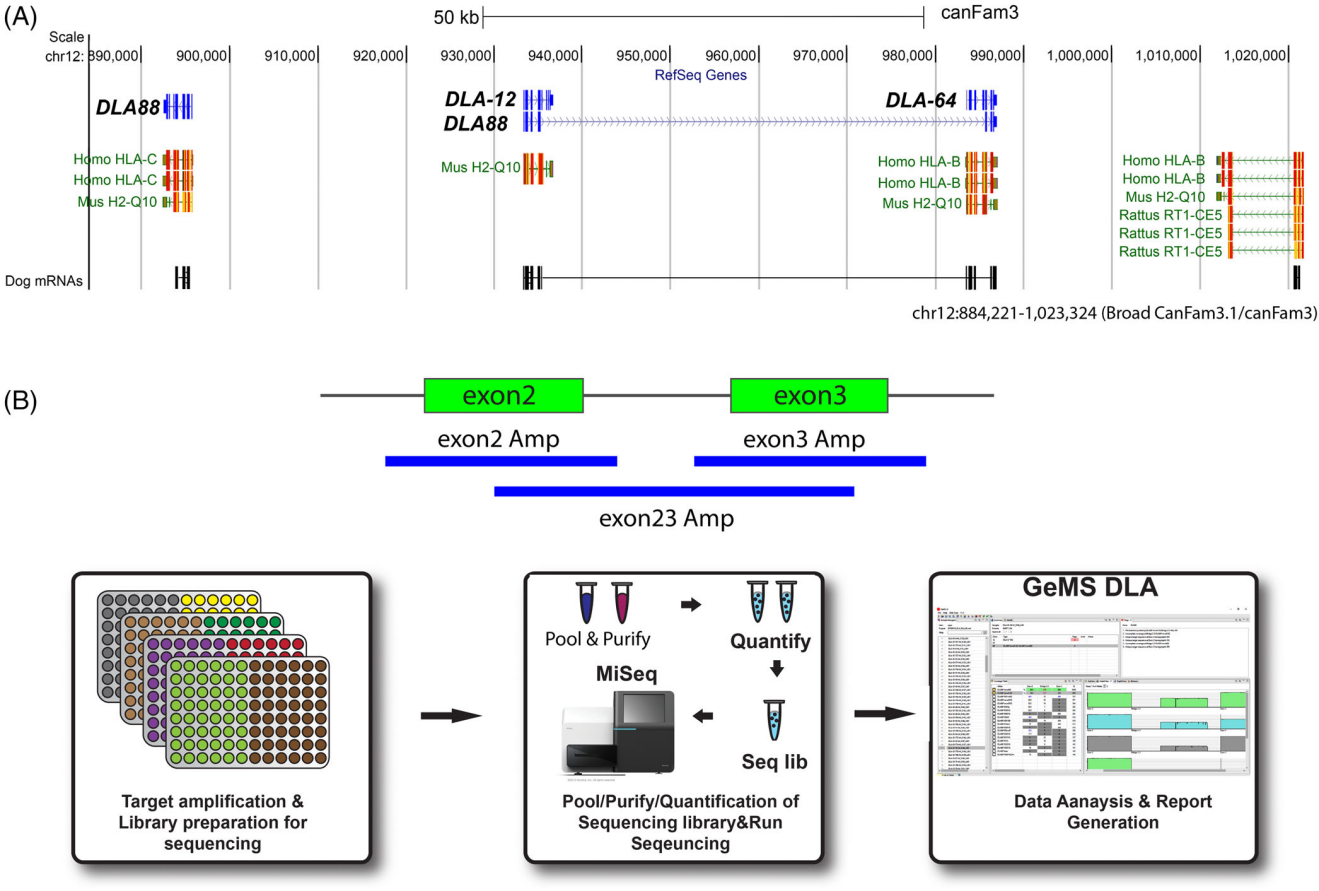
Strategy for next generation sequence‐based typing of DLA‐88. (A) The genomic region of the canine major histocompatibility complex (MHC) with the positions of the DLA class I genes indicated in blue with annotated names. The exon/intron structures of each gene are depicted with raised bars (exons) and lines (introns). Human and rodent class I genes are similarly depicted with red bars and annotated. The overlay of DLA‐88 and DLA‐12 genes is intended to designate the different haplotypes identified putatively generated via gene conversion.^28^ The relative placements of genes from other species were taken from the UCSC Genome Browser on Dog September 2011 (Broad CanFam3.1/canFam3) Assembly. (B) The NGS workflow for DLA‐88 exons 2 and 3 is indicated with the positions of the PCR amplicons (blue bars) indicated beneath the exon/intron structure. The laboratory workflow is indicated in three sequential boxes from left to right, from target amplification through purification and sequencing, to data analysis. Both the laboratory workflow and computational analysis were designed in accord with previously established methods for HLA analysis^10^, ^29^

Exons 2 and 3 of the DLA‐88 gene, as well as an overlapping region spanning intron 2, were sequenced and phased using a targeted amplicon approach similar to that previously described for the HLA system.^10^ After identifying target regions, primers were designed to amplify fragments ranging from 300 to 500 bp in length (Figure 1, Supplemental Figure S1). Multiple primer sets were designed where polymorphisms have been detected at the primer sites (Supplemental Table S1). We used two different sets of primers for each exon to lower the probability of allelic drop out caused by unknown variants at the amplification primer binding sites.

Amplicons were extended to include barcode sequences (uniquely identifying each genomic sample). The S1 and S2 buffers and the indexing system were supplied by Scisco Genetics Inc., and the basic protocol including PCR reaction cycling was identical to that for HLA typing methods detailed on the product insert for the ScisGo®‐HLA‐v6 typing kit (Scisco Genetics Inc., Seattle WA, www.sciscogenetics.com/pages/kits.html). This system for barcoding with custom primers was adapted from the commercial chimerism kit from Scisco Genetics Inc. (ScisGo®‐HLA‐v5) after exchanging the HLA primer reactions contained in that kit for the DLA‐88 primers. After assembly, the protocol provided for the HLA system was followed precisely using the five DLA‐88 primer sets as five individual amplicons.

With target generation and library preparation complete, all 96 bar‐coded samples for each of 5 individual reactions were pooled together into individual tubes and column filtered using a Select‐a‐Size DNA Clean and Concentrator (Zymo Research, Irvine, CA) to remove short (<200 bp) DNA. Filtered pools were quantified using the Quant‐iT™ PicoGreen dsDNA Assay Kit (Life Technologies, Carlsbad, CA). Quantified pools were combined in equal molar amounts to form a single pool, which was then diluted and denatured in accordance with the standard MiSeq loading protocol (Illumina, San Diego CA). Illumina version 2 chemistry was used for 500‐cycle, paired‐end sequencing. Genotyping was performed using alignment and type selection methods originally developed for HLA^10^ and extended for this study. Software for derivative data analysis used an adaptation of GeMS‐UI (https://sciscogenetics.com/pages/software.html) (Scisco Genetics Inc., Seattle WA). Read depths for exons 2 and 3 were greater than 1000 reads per animal whereas exons 2–3 overlapping sequence read depth was on average 225 reads per dog. Database lookup was also used to confirm phase of exons 2 and 3 via comparison of exon sequences with known alleles of DLA‐88 and other class I genes in the IPD DLA database. The system did not attempt to establish typing for DLA‐12. For example, if exon 3 sequences derive from DLA‐88*00402 and exon 2 from DLA‐88*00402 (and possibly DLA‐12*1 since the two loci have identical exon 2 sequences) then the database lookup logic will assign DLA‐88*00402 as the DLA‐88 genotype. However, the logic does leave open the possibility that exon 3 is from a known allele, while exon 2 has shown an allele drop off.

As controls for PCR cross contamination for NGS, each 96‐sample reaction plate contained at least 2 negative wells (all reaction components without genomic DNA) and 2 positive samples with known DLA types, previously established by the Sanger method. Appropriately, negative and positive results indicated that the reagents were uncontaminated and functioning as designed. To control for sample‐to‐sample contamination, each reaction plate was assembled in duplicate, and the typing results compared between duplicate samples. Any inconsistency in typing results would be expected to be derived from sample‐to‐sample contamination. All results included in this report were derived from experiments that satisfied these controls.

Final allele calls included manual editing of a subset of the samples where the automated processes yielded ambiguous results, and visual inspection was carried out for all results examining read depth and call consistency. All new alleles discovered in this study have been submitted to GenBank and IPD‐MHC with accession numbers as listed in Table 1.

**TABLE 1:** Summary of DLA alleles identified in this study

Accession	Allele	Status	No. of dogs	Accession	Allele	Status	No. of dogs
DLA07967	DLA‐88*001:01	Public	1	DLA08292	DLA‐88*043:04	Public	1
DLA08080	DLA‐88*002:01	Public	61	***MN188078.1***	DLA‐88*044m008	***Not assigned***	***1***
***DLA08323***	DLA‐88*002:02	***Public***	***3***	DLA08237	DLA‐88*045:01	Public	5
***MK617598.1***	DLA‐88*002m04501	***Not assigned***	***1***	DLA08158	DLA‐88*045:02	Public	9
DLA08269	DLA‐88*003:02	Public	22	DLA08169	DLA‐88*046:01	Public	1
DLA08299	DLA‐88*003:03	Public	1	***DLA08344***	DLA‐88*046:02	***Public***	***7***
DLA08113	DLA‐88*004:02	Public	46	***MK617597.1***	DLA‐88*047:01m	***Not assigned***	***2***
***DLA08325***	DLA‐88*004:03	***Public***	***13***	DLA08168	DLA‐88*049:01	Public	2
DLA07975	DLA‐88*005:01	Public	28	DLA08301	DLA‐88*049:02	Public	4
***MK617612.1***	DLA‐88*00501m	***Not assigned***	***1***	***DLA08342***	DLA‐88*049:03	***Public***	***3***
DLA07988	DLA‐88*006:01	Public	40	***DLA08339***	DLA‐88*050:02	***Public***	***5***
DLA08300	DLA‐88*006:02	Public	8	***DLA08347***	DLA‐88*050:03	***Public***	***8***
***MK617588.1***	DLA‐88*006m47	***Not assigned***	***3***	DLA08206	DLA‐88*051:01	Public	25
DLA08005	DLA‐88*007:01	Public	1	DLA08287	DLA‐88*052:01	Public	5
DLA08312	DLA‐88*007:02	Public	20	DLA08307	DLA‐88*052:02	Public	4
DLA08023	DLA‐88*008:01	Public	3	DLA08290	DLA‐88*054:01	Public	6
***DLA08343***	DLA‐88*008:02	***Public***	***4***	DLA08295	DLA‐88*056:01	Public	7
DLA08063	DLA‐88*010:01	Public	40	DLA08296	DLA‐88*057:01	Public	2
DLA08165	DLA‐88*012:01	Public	55	DLA08298	DLA‐88*058:01	Public	6
DLA08305	DLA‐88*013:02	Public	12	DLA08303	DLA‐88*060:01	Public	2
DLA08288	DLA‐88*014:01:02	Public	10	DLA08304	DLA‐88*060:02	Public	11
DLA08078	DLA‐88*015:01	Public	1	***DLA08327***	DLA‐88*060:03	***Public***	***3***
DLA08081	DLA‐88*016:02	Public	3	DLA08306	DLA‐88*061:01	Public	1
DLA08253	DLA‐88*016:03	Public	11	DLA08309	DLA‐88*062:01	Public	4
DLA08224	DLA‐88*016:04	Public	11	DLA08310	DLA‐88*063:01	Public	1
DLA08319	DLA‐88*016:05	Public	3	DLA08311	DLA‐88*064:01	Public	2
***MK617573.1***	DLA‐88*01601N25m	***Not assigned***	***8***	DLA08314	DLA‐88*065:01	Public	7
DLA08082	DLA‐88*017:01	Public	20	***DLA08316***	DLA‐88*067:01	***Public***	***9***
DLA08297	DLA‐88*019:02	Public	2	***DLA08334***	DLA‐88*067:02	***Public***	***6***
DLA08278	DLA‐88*021:01	Public	1	***DLA08326***	DLA‐88*069:01	***Public***	***6***
DLA08086	DLA‐88*022:01	Public	9	***DLA08336***	DLA‐88*069:02	***Public***	***6***
DLA08318	DLA‐88*024:02	Public	20	***DLA08332***	DLA‐88*072:01	***Public***	***16***
DLA08321	DLA‐88*024:03	Public	6	***DLA08333***	DLA‐88*073:01	***Public***	***4***
DLA08089	DLA‐88*025:01	Public	2	***DLA08338***	DLA‐88*075:01	***Public***	***3***
***MK617611.1***	DLA‐88*02501m	***Not assigned***	***1***	***DLA08340***	DLA‐88*076:01	***Public***	***4***
DLA08322	DLA‐88*026:02	Public	4	***DLA08341***	DLA‐88*077:01	***Public***	***3***
***MT543269.1***	DLA‐88*02601m2	***Not assigned***	***1***	***DLA08346***	DLA‐88*078:01	***Public***	***6***
DLA08093	DLA‐88*028:01	Public	32	***DLA08348***	DLA‐88*079:01	***Public***	***4***
DLA08205	DLA‐88*028:03	Public	7	DLA08108	DLA‐88*501:01	Public	9
DLA08294	DLA‐88*028:05	Public	13	DLA08354	DLA‐88*501:02	Public	14
DLA08308	DLA‐88*028:06	Public	2	DLA08109	DLA‐88*502:01	Public	2
DLA08313	DLA‐88*028:07	Public	4	DLA08115	DLA‐88*507:01	Public	2
***MK617610.1***	DLA‐88*02801m2	***Not assigned***	***2***	DLA08116	DLA‐88*508:01	Public	31
DLA08156	DLA‐88*029:01	Public	57	DLA08357	DLA‐88*508:03	Public	2
DLA08320	DLA‐88*029:02	Public	1	***DLA08349***	DLA‐88*511:02	***Public***	***4***
***DLA08345***	DLA‐88*030:02	***Public***	***3***	***DLA08358***	DLA‐88*512:01	***Public***	***6***
***MK617613.1***	DLA‐88*03001m	***Not assigned***	***1***	***DLA08359***	DLA‐88*513:01	***Public***	***5***
DLA08194	DLA‐88*032:01	Public	2	***MK617607.1***	DLA‐88*AmN16	***Not assigned***	***1***
DLA08204	DLA‐88*032:02	Public	2	***MN188080.1***	DLA‐88*Bm28	***Not assigned***	***1***
DLA08097	DLA‐88*034:01	Public	7	***MK617579.1***	DLA‐88*N16m1	***Not assigned***	***1***
***MK617595.1***	DLA‐88*03401m	***Not assigned***	***2***	***MK617594.1***	DLA‐88*N2m036	***Not assigned***	***2***
DLA08098	DLA‐88*035:01	Public	4	***MK617606.1***	DLA‐88*N31mN40	***Not assigned***	***1***
DLA08198	DLA‐88*036:01	Public	1	***MK617592.1***	DLA‐88*N32m1	***Not assigned***	***2***
DLA08291	DLA‐88*036:02	Public	11	***MK617590.1***	DLA‐88*N39m46	***Not assigned***	***1***
DLA08100	DLA‐88*038:01	Public	2	***MK617603.1***	DLA‐88*N40m1	***Not assigned***	***1***
DLA08101	DLA‐88*039:01:01	Public	4	***MK617602.1***	DLA‐88*N45m49	***Not assigned***	***1***
***MK617609.1***	DLA‐88*03901m	***Not assigned***	***1***	***MK617600.1***	DLA‐88*N46mN25	***Not assigned***	***1***
DLA08135	DLA‐88*040:01	Public	4	***MK617585.1***	DLA‐88*N5m	***Not assigned***	***3***
DLA08103	DLA‐88*041:01	Public	4	***MT597426.1***	DLA‐88*N7m	***Not assigned***	***1***
DLA08286	DLA‐88*042:02	Public	3	***MK617593.1***	DLA‐88*N7m1	***Not assigned***	***2***
***MN167163.1***	DLA‐88*042m40	***Not assigned***	***1***	***LC130518.1***	DLA‐88*novel25	***Not assigned***	***9***
DLA08284	DLA‐88*043:02	Public	31	***MW139898***	DLA‐88*novel25V	***Not assigned***	***2***
***DLA08285***	DLA‐88*043:03	***Public***	***4***				

*Note*: Bold/Italic/underlined marks new alleles detected in this study.

*Note*: DLA‐88L alleles (as defined by Miyamae et al.^28^) are highlighted in green.

### 2.3 Verification of NGS results by dye terminator / capillary gel sequencing

Amplification of the polymorphic region of *DLA‐88* (exons 2 and 3) was performed as described.^23^, ^24^ Briefly, a 1.2 kilobase fragment containing exons 2 and 3 (Supplemental Figure S1) was amplified and the gel‐purified product was cloned with the Invitrogen TA cloning kit according to manufacturer's instructions (ThermoFisher, Waltham, MA). Both the PCR product and 8–12 clones were then sequenced directly by conventional fluorescent dideoxynucleotide chain termination reactions and capillary gel fragment analysis. In cases where this approach did not detect alleles called by NGS, 20 additional clones were sequenced. New alleles were defined according to the criteria of (i) DLA‐88‐homologous sequences, (ii) identified in two or more dogs by both methods and, (iii) not present in either the IPD‐MHC or GenBank databases.

## 3 RESULTS

### 3.1 DLA typing by NGS sequencing

The canine DLA class I region contains at least three highly homologous DLA class I genes, including DLA‐88, DLA‐12 and DLA‐64, in addition to haplotype variation that includes a duplication of DLA‐88 where DLA‐12 is replaced by a second DLA‐88 locus. This duplicated DLA‐88 has been reported as DLA‐88‐like (DLA‐88L) residing at the neighboring DLA‐12 locus, possibly resulting from a gene conversion event between these genes^29^ (see Figure 1A for derivative depiction). Miyamae et al.^28^ reported 10 DLA‐88 alleles (016:03, 016:04, 016:05, 017:01, 024:02, 024:03, 026:02, 029:01, 029:02, and novel_30) that were assigned as DLA‐88L. Throughout this paper we included alleles of DLA‐88L as part of a larger group of DLA‐88 alleles when using numerical references to DLA‐88 alleles (numbers of alleles) and such references should be considered to include both loci/alleles. In order to develop and execute a next generation sequencing platform for the canine DLA‐88 locus, an amplicon‐based approach was designed using a simplified version of the previously described amplicon‐based HLA typing method.^10^, ^29^ The hypervariable regions (HVRs) of DLA‐88 were targeted using amplicons specific for each of the full exons 2 and 3 and a phasing amplicon (exon 23 Amp) including overlapping sequences from exons 2 and 3 and the intervening intron 2 sequence (Figure 1B). Due to homology among DLA class I genes, it was not possible to exclude amplification of sequence reads from exon 2 of a subset of DLA‐12 alleles. However, it was possible to segregate these reads at the data analysis stage using the known panel of DLA‐12 alleles, enabling independent analysis of DLA‐88 sequences. This was possible due to the divergence of DLA‐12 exon 3 and flanking intron sequences, which differ significantly from DLA‐88, allowing both discrimination of exon 3 by the amplification primers and by direct examination of the DLA‐88 and DLA‐12 exon 3 sequences. The possibility that a novel exon 2 read belonged to a novel DLA‐12 rather than as assigned to a DLA‐88 was ruled out for all the novel alleles detected by both Sanger and NGS and for those detected only by NGS by a corresponding exon23 Amp sequence.

This approach was initially tested using a panel of 286 canine DNAs derived from a total of 86 dog breeds, although sample specific breed assignment was uncertain. In order to obtain breed‐specific DLA‐88 frequency NGS typing data, the method was applied to 96 additional samples with assigned breeds consisting of a DNA panel of 48 Great Danes and 48 German Shepherds that had been DLA typed by the Sanger Sequencing method in the FHCC DLA typing shared resource. Results of this analysis are presented in Supplemental Table S4. The overall performance of the NGS approach was robust, with very consistent exons 2 and 3 read depths over 1000 reads in a single MiSeq run of 96 samples, suggesting as many as fourfold more samples could be loaded on a single run. The read depth of phasing amplicon (exon 23 amp) was lower (~225 reads per dog) and more variable sample to sample, but nonetheless sufficiently robust (>100 reads) to establish the phase of exons 2 and 3 in most cases. A likely cause of sample‐to‐sample variation was primer drop down due to variability in exons 2 and 3 not accounted for in the initial phasing primer design. This explanation is supported by the number of new alleles discovered in this report as described below. However, for all samples reported here, in 20 cases where the sequence of phasing amplicons was not informative, the exon 2/exon 3 data were sufficient to unambiguously identify an allele by data lookup using the expanded DLA‐88 database. The expanded database consisted of all reported DLA‐88 alleles in IPD DLA database and Genbank including newly identified alleles from this study (Supplemental Table S2). In fact, in all cases it was possible to unambiguously assign phase using only exon 2 and exon 3 sequences via database lookup with the exon23 phasing Amp uniformly confirming the assignment.

We used only the assembled sequences of exon 2 and exon 3 with at least 100 reads at all positions for DLA typing. All new alleles detected in this study had at least 1 base change (average 5.3 base changes) compared with DLA‐88 alleles or sequences in either the IPD‐DLA database or GenBank. For example, DLA‐88*072:01 detected in 15 dogs (Table 1) is different at 5 different nucleotide positions from the closest allele (DLA‐88*008:01). A total of 125 unique alleles were detected in this study including 51 new alleles, of which 24 have been officially assigned in the current IPD database with 27 unassigned as of this writing (Table 1, Supplemental Table S2).

A summary of allele calls from NGS analysis is shown in Table 2. Two significant findings arise from this data. First, a large fraction of the samples yielded 3 to 5 distinct DLA‐88 alleles, rather than the expected 1–2 for a single‐locus gene. In that regard, at least 3 alleles were detected in 134 dogs (35%), at least 4 alleles in 38 dogs (10%), and 5 alleles in 4 dogs (1%). When DLA‐88L alleles are present, the carrier dog has two copies of DLA‐88 and 1–2 copies of DLA‐88L but lacks one or both copies of DLA‐12. We found at least one of these DLA‐88L alleles in 121 (32%) of the dogs in this study (Table 2).

**TABLE 2:** Number of DLA‐88 alleles in individual dogs

DLA‐88 alleles present (including both DLA‐88 & DLA‐88L)	DLA‐88(+)/DLA‐88L(−)/DLA‐12(+)	(%)	DLA‐88(+)/DLA‐88L(+)/DLA‐12(+)	(%)	DLA‐88(+)/DLA‐88L(+)/DLA‐12(−)	(%)	Total
2	196	95.1	3	1.5	7	3.4	206
3	57	42.5	76	56.7	1	0.7	134
4	8	21.1	18	47.4	12	31.6	38
5	0	0.0	0	0.0	4	100.0	4

The second significant finding was a high rate of new‐allele discovery. A total of 51 unique and previously unknown DLA‐88 allele sequences were detected among the 382 DLA typed samples (Table 1). These new allele candidates differed from their closest match among reported alleles by 1–12 bases. In every case, the substitutions resulted in at least one change in the predicted amino acid sequence. New alleles were found at frequencies of 1 to 16 dogs among the 382 called. A total of 34 new‐allele candidates were observed in two or more dogs (Supplementary Table S2). A measurement of allelic frequencies in specific breeds was examined using Great Danes and German Shepherds, essentially replicating those found in Ross et al.^27^ and Miyamae et al.^28^ (Supplemental Table S4).

### 3.2 Verification of new alleles by Sanger sequencing

Selected animals with novel alleles detected by NGS were further characterized for DLA‐88 variation using ABI dye terminator technology (Sanger sequencing) over exons 2 and 3 with phasing deduced from direct sequencing of the PCR products and derivative clones.^24^ None of the amplification or sequencing primer sequences used in the Sanger sequencing were common to those designed for the NGS system (Supplemental Table S1). We focused on carriers of alleles detected in two or more animals by NGS. A total of 63 carriers were analyzed, collectively including 29 novel alleles detected in at least two animals. An additional seven single‐carrier alleles also occurred in this group.

Direct sequencing of PCR products and deducing alleles from ambiguous sequence traces, failed to identify the majority of the alleles detected by NGS (data not shown). Cloning the PCR product and sequencing eight derived clones (the standard protocol for typing) still missed at least one allele in more than half of the animals, presumably due to allele drop‐off during the PCR. In these cases, the number of sequenced clones was increased to 20 or more.

Results of the two methods are compared in Table 3. Of the 63 animals analyzed, 39 (62%) yielded fully concordant results by both methods. In 23 dogs (37%), the Sanger method failed to detect at least one allele called by NGS. Conversely, only one allele call (1.6%) by the Sanger method was missed by NGS. All target exon sequences for individual samples generated by both methods for individual samples were perfectly matched with no SNP or InDel discrepancies.

**TABLE 3:** Comparative identification of novel DLA‐88 alleles by NGS and Sanger approaches^a^

Dog	Allele calls common to both methods					Additional method‐specific Allele calls		DLA‐12 detected by NGS
	Allele 1	Allele 2	Allele 3	Allele 4	Allele 5	NGS‐exclusive Calls	Sanger‐exclusive Calls	
17	DLA‐88*005:01	DLA‐88*067:02	DLA‐88*078:01					Yes
45	DLA‐88*072:01	DLA‐88*508:01						Yes
50	DLA‐88*002:02	DLA‐88*501:02						Yes
90	DLA‐88*050:02	DLA‐88*N16m1	DLA‐88*501:02			DLA‐88*501:01		Yes
99	DLA‐88*032:02	DLA‐88*047:01m	DLA‐88*077:01					Yes
100	DLA‐88*006:01	DLA‐88*072:01						Yes
101	DLA‐88*016:03	DLA‐88*067:02	DLA‐88*073:01	DLA‐88*078:01				Yes
109	DLA‐88*006:01	DLA‐88*008:02	DLA‐88*042:02					Yes
156	DLA‐88*002:02	DLA‐88*029:01				DLA‐88*028:01		Yes
170	DLA‐88*029:01	DLA‐88*N32m1				DLA‐88*004:02		Yes
171	DLA‐88*005:01	DLA‐88*N5m				DLA‐88*007:02		Yes
173	DLA‐88*028:01	DLA‐88*029:01	DLA‐88*N5m					No
385	DLA‐88*076:01							Yes
412	DLA‐88*043:02	DLA‐88*067:02	DLA‐88*078:01					Yes
417	DLA‐88*006:01	DLA‐88*047:01m	DLA‐88*077:01					Yes
427	DLA‐88*002:01	DLA‐88*N5m						Yes
435	DLA‐88*024:02	DLA‐88*038:01	DLA‐88*050:02					Yes
436	DLA‐88*043:03	DLA‐88*508:01						Yes
472	DLA‐88*054:01	DLA‐88*069:02				DLA‐88*069:01		Yes
505	DLA‐88*005:01	DLA‐88*024:02	DLA‐88*050:02			DLA‐88*007:02		No
508	DLA‐88*036:02	DLA‐88*069:02				DLA‐88*079:01		Yes
530	DLA‐88*002:01	DLA‐88*029:01				DLA‐88*N32m1		Yes
688	DLA‐88*076:01	DLA‐88*507:01						Yes
689	DLA‐88*016:04	DLA‐88*024:02	DLA‐88*050:03	DLA‐88*512:01		DLA‐88*006m47		No
703	DLA‐88*052:01	DLA‐88*069:02				DLA‐88*069:01		Yes
746	DLA‐88*067:01					DLA‐88*003:02		No
750	DLA‐88*002:01	DLA‐88*046:02					DLA‐88*079:01	Yes
753	DLA‐88*004:02	DLA‐88*016:03	DLA‐88*073:01					Yes
766	DLA‐88*003:03	DLA‐88*072:01						Yes
793	DLA‐88*067:01					DLA‐88*003:02		No
798	DLA‐88*024:02	DLA‐88*508:03	DLA‐88*513:01			DLA‐88*050:03		Yes
808	DLA‐88*006:01	DLA‐88*01601N25m						Yes
831	DLA‐88*013:02	DLA‐88*024:02	DLA‐88*075:01					Yes
843	DLA‐88*006:01	DLA‐88*008:02	DLA‐88*042:02					Yes
872	DLA‐88*01601N25m	DLA‐88*051:01						Yes
873	DLA‐88*01601N25m	DLA‐88*049:03						Yes
875	DLA‐88*N7m1	DLA‐88*043:02						Yes
887	DLA‐88*508:01	DLA‐88*N2m036						Yes
969	DLA‐88*072:01					DLA‐88*046:02		Yes
979	DLA‐88*006:01	DLA‐88*077:01				DLA‐88*006:02		Yes
986	DLA‐88*004:02	DLA‐88*016:03	DLA‐88*073:01					Yes
991	DLA‐88*006:01	DLA‐88*049:03				DLA‐88*006:02		Yes
1018	DLA‐88*076:01	DLA‐88*507:01						Yes
1105	DLA‐88*008:02	DLA‐88*035:01	DLA‐88*042:02					Yes
1117	DLA‐88*014:01:02	DLA‐88*067:01	DLA‐88*065:01					Yes
1128	DLA‐88*N39m46					DLA‐88*042 m40		Yes
1132	DLA‐88*016:04	DLA‐88*024:02	DLA‐88*050:03	DLA‐88*512:01		DLA‐88*006m47		No
1134	DLA‐88*046:02	DLA‐88*051:01						Yes
1136	DLA‐88*508:01	DLA‐88*513:01						Yes
1138	DLA‐88*508:01	DLA‐88*513:01						Yes
1142	DLA‐88*005:01	DLA‐88*024:02	DLA‐88*075:01			DLA‐88*Bm28		No
1146	DLA‐88*01601N25m	DLA‐88*049:03				DLA‐88*N7m1		Yes
1155	DLA‐88*069:01	DLA‐88*508:01				DLA‐88*AmN16		Yes
1158	DLA‐88*012:01	DLA‐88*N2m036						Yes
1159	DLA‐88*002:02	DLA‐88*046:02						Yes
1161	DLA‐88*006:01	DLA‐88*024:02	DLA‐88*075:01					Yes
1171	DLA‐88*034:01	DLA‐88*069:02	DLA‐88*079:01					Yes
1178	DLA‐88*043:03	DLA‐88*060:03						Yes
1189	DLA‐88*016:04	DLA‐88*024:02	DLA‐88*050:03	DLA‐88*512:01		DLA‐88*006m47		No
1210	DLA‐88*004:02	DLA‐88*03401m						Yes
1211	DLA‐88*002:01	DLA‐88*03401m						Yes
1214	DLA‐88*043:03	DLA‐88*060:03						Yes
1234	DLA‐88*060:03	DLA‐88*501:02				DLA‐88*501:01		Yes

^a^ A 63 dogs carrying new alleles according to NGS, were re‐tested by Sanger sequencing.

Even though the Sanger method missed a significant number of alleles detected by NGS, we were able to verify 25 novel alleles based on detection in at least two dogs by both methods (Supplemental Table S3).

## 4 DISCUSSION

### 4.1 DLA‐88 typing by NGS

In this report, we designed and evaluated an NGS approach to DLA‐88 typing in the dog, based on methods we had established previously for HLA typing.^10^, ^28^, ^29^ This DLA typing NGS system showed higher accuracy and sensitivity than the Sanger approach when considering the number of alleles that went undetected by the Sanger method. In addition, the NGS system demonstrated lower cost and higher sample throughput over the Sanger method where the latter could extend the time for typing from start to finish by up to 3 weeks for as few as 20 samples while DLA NGS could be used to complete typing of 96 samples in 4 days.

Using this method to type 382 dogs, we found unexpectedly high frequencies of multiple allele carrier dogs (>2 DLA‐88 alleles) reflecting gene copy number variation, as well as a high discovery frequency of novel DLA‐88 alleles. The NGS method was far more sensitive than Sanger, missing only one call (1.6%) while the Sanger method missed 37% of the allele calls made by NGS (Table 3). The missed call rate was much higher for the Sanger method due to allele amplification bias that was not present in the NGS method at similar levels, in part due to the application of multiple amplicons based on available known allele data as well as the higher read depth and inherent clonality of NGS compared to the Sanger sequencing approach. At the same time, the NGS method will require further refinement to allow detection of all alleles as, indeed, one was missed in this data set.

### 4.2 Dogs with multiple‐copies of DLA‐88

Previous reports have described dogs carrying more than two DLA‐88 alleles,^22^, ^30^ suggesting that the DLA‐88 gene had been duplicated in those cases. Consistent with these findings, Miyamae et al.^28^ recently described a subset of DLA‐88L alleles that are located at the DLA‐12 locus, presumably due to a gene conversion event between these two genes. The sequences of the DLA‐88L alleles are more similar to DLA‐88 than DLA‐12, especially in the antigen‐binding pocket regions encoded by exons 2 and 3 and have similar expression profiles. Therefore, the DLA‐88L alleles are probably best considered as functional DLA‐88 alleles in the context of tissue matching. Dogs that carry these DLA‐88L alleles express 3 or 4 copies of DLA‐88 and are missing copies of the DLA‐12 gene. Altogether, 186 dogs (49%) either carried a DLA‐88L allele, or carried at least three detectable DLA‐88 alleles, or both (Supplemental Figure S2). Thus, about half of domestic dogs may carry 3 or 4 copies of DLA‐88 (including DLA‐88L). This frequency of multiple allele dogs is significantly higher than that reported by Miyamae et al.^28^ (105 of 404 unrelated dogs or 26%).

The DLA‐88L alleles previously reported do not account for all the multi‐allele dogs in this study. Only 63% of multi‐alleles dogs carry a known DLA‐88L allele (Table 2). This may indicate that additional DLA‐88L alleles have not yet been identified as such, and that some of the new alleles identified here may fit into that category. However, our data does not address the location of the extra DLA‐88 alleles and cannot yet assign any of them to the subset of DLA‐88L alleles. Alternatively, a second duplication event may have generated another family of DLA‐88L alleles that remain to be characterized. Such an event would explain the cases in which five alleles were detected. The possibility of sample cross‐contamination in at least some cases of multiple‐allele detection is unlikely as independent isolates from the same animal yielded identical results in all cases reported here. However, other contamination of canine genomic DNA (besides cross‐contamination, i.e., from previous blood transfusion or fetal‐maternal microchimerism) could play a confounding role in the discovery of “extra” DLA‐88 copies. We consider these possibilities unlikely as the dogs used in this study were not to our knowledge subjected to prior blood transfusion and relative levels of DNA contamination due to fetal‐maternal chimerism should be low enough to avoid detection of contaminating genomic DNA in PCR amplification.

It should be noted that much of the validation of these multiple allele cases by Sanger sequencing occurred only because we already knew what to look for based on the NGS findings. We usually type animals with the expectation of 1 or 2 alleles. Under this assumption, minor secondary peaks in direct sequencing are often ignored as probable noise. If cloning is deemed necessary, relatively few clones are sequenced (typically eight), based on the same assumption. The deep sequencing by NGS has a clear advantage over Sanger sequencing in detection of multiple allele dogs. This apparent frequent multiple allele condition in domestic dogs could complicate DLA matching for tissue transplantation. If typing is done by Sanger sequencing, under the assumption of two copies of DLA‐88, vital mismatches could be missed that could lead to unpredicted (and unexplained) rejection.

### 4.3 New DLA‐88 alleles

The frequency of new allele discovery was high. A total of 51 novel alleles of DLA‐88 are reported here, 25 of which were verified in at least two dogs by both methods. Since all of the 51 new alleles reported here encode a unique amino acid sequence within the antigen‐binding pocket, they likely represent functionally unique alleles. The rate of new allele discovery in this study (51 novel candidate alleles in 382 dogs = 13%), suggests that the real complexity of the DLA‐88 allele set in the canine population is several fold higher than the known complexity so far, consistent with conclusions from prior studies.^24^, ^28^ The NGS approach described here is expected to accelerate the discovery of novel DLA‐88 alleles with a much higher discovery rate, and a much lower cost than could be expected from Sanger sequencing.

Although DLA‐88 allelic heterogeneity is evidently greater than previously described, it should be pointed out that it has yet to be established that the new alleles and multiple loci play a role in matching for transplantation. It is possible that some of the new alleles reported are null alleles (i.e., not expressed). The NGS sequencing reported here only provided partial sequencing from genomic DNA, the necessary analysis for full‐length translatable sequences has not been completed, nor has the DLA transcriptome been profiled. While NGS has clear advantages for typing, it's important to point out that matching at the DLA‐88L (or even a third locus) may be excessive for biologically‐irrelevant alleles. Further profiling work to define expression patterns will be needed to improve NGS relevance in the context of transplantation. In that regard, the fact that NGS revealed the existence of potential DLA contributors that the Sanger approach missed, further recommends routine use of NGS in dog transplantation to help drive such studies.

## AUTHOR CONTRIBUTIONS

Chul‐Woo Pyo: Designed assays and performed NGS sequence analysis, coauthored and edited the manuscript. Michael A. Harkey: Designed and performed conventional sequence analysis, coauthored and edited the manuscript. Beverly Torok‐Storb: Provided scientific and laboratory support and edited the manuscript. Rainer Storb: Designed and initiated the study and edited the manuscript. Alexander Thomas: Performed NGS sequence analysis. Ruihan Wang and Wyatt C. Nelson: Designed computational adaptations for DLA NGS sequence analysis derivative from an existing cloud based and local installation HLA framework. Daniel E. Geraghty: Designed the overall workflow, coordinated laboratory and analysis activities, and coauthored and edited the manuscript.

## FUNDING INFORMATION

This work was supported by grant U54 DK106829 (BTS) from the National Institute of Diabetes and Digestive and Kidney Diseases (NIDDK) of the National Institutes of Health (NIH), Bethesda, MD, USA.

## CONFLICT OF INTEREST

Daniel E Geraghty is founder and co‐owner of Scisco Genetics Inc. (Seattle, WA). Chul‐Woo Pyo, Ruihan Wang, and Wyatt C Nelson are employed in part by Scisco Genetics Inc. The remaining authors have no financial relationships that can present a potential conflict of interest in these studies.

## Supporting information


**Appendix S1** Supporting Information.

## Data Availability

The data that support the findings of this study are openly available in genbank at https://www.ncbi.nlm.nih.gov/genbank/.
