# Supplementary material for: Genotyping of canine MHC gene DLA‐88 by next‐generation sequencing reveals high frequencies of new allele discovery and gene duplication
Source: HLA. 2022 Aug 9;100(5):479–90. doi: 10.1111/tan.14752 (PMC9563979; doi:10.1111/tan.14752)
Supplement: Supplementary file 1 — Appendix S1 Supporting Information. [file TAN-100-479-s001.pdf]

SUPPLEMENTAL DATA

Supplemental Table 1. Primer sequences used for amplification of exons 2 and 3 of DLA-88 in the NGS and Sanger protocols.*

Region		Forward		Reverse	
		**name**	**sequence**	**name**	**sequence**
exon2	set1	ex2-v2-F	GGGAGGGGGTCGGGCGGGGTCTCAC	ex2-v2-R	CGGCCGGTCGGGGGGATGGGGGT
	set2	ex2-v3-F1	GGTCTCAGCCCTCGGCTCCGCAG	ex2-v3-R1	GGCGACCCGGCCGGTCGGGGGGAT
		ex2-v3-F2	GGTCTCACCCGTCGGCTCCGCAG	ex2-v3-R2	GGCGACCCGGCCGGTCGGGGGCAT
		ex2-v3-F3	GGTCTCACCCGTCGGCCCCGCAG	ex2-v3-R3	GGCGACCCGGCCGGCCGGGGGGAT
exon3	set1	ex3-v1-F	CTCTTTGGACTGATTGGCGGCCTGTC	ex3-v1-R	CTGGAGGGAAGCCCTCCCTAGTGG
	set2	ex3-v2-F	CTTTCTCTTTGGACTGATTGGCGGC	ex3-v2-R	TTCCCTTCCTGGAGGGAAGCCCTCCCT
Bridge-ex23	set1	ex23-v1-F1	TGGAGCAGGAGGGGCCGGAGTATTG	ex23-v1-R1	CGCACGTCGTCTCCAGGTAGTTC
		ex23-v1-F2	TGGGGCAGGAGGGGCCGGAGTATTG	ex23-v1-R2	CGCACGTCATCTCCAGGTAGTTC
				ex23-v1-R3	CGCACGTCGTCTCCAGGTAGCTC
Sanger	ex23	2924	GCGGCGACGGCCAGTGTCCCCGGAG	2928	GACCCTGAGTCCATATTCCCTTCC

*The detailed method is described by Venkataraman et al.[24]

SUPPLEMENTAL TABLE 2. Complete DLA-88 allele list and frequencies found by NGS

Accession (IPD/GenBank)	Name	Status	Number of cases
DLA08332	DLA-88*072:01	public	16
DLA08325	DLA-88*004:03	public	13
DLA08316	DLA-88*067:01	public	9
DLA08347	DLA-88*050:03	public	8
MK617573.1	DLA-88*01601N25m	Not assigned	8
DLA08344	DLA-88*046:02	public	7
DLA08334	DLA-88*067:02	public	6
DLA08326	DLA-88*069:01	public	6
DLA08336	DLA-88*069:02	public	6
DLA08346	DLA-88*078:01	public	6
DLA08358	DLA-88*512:01	public	6
DLA08339	DLA-88*050:02	public	5
DLA08359	DLA-88*513:01	public	5
DLA08343	DLA-88*008:02	public	4
DLA08285	DLA-88*043:03	public	4
DLA08333	DLA-88*073:01	public	4
DLA08340	DLA-88*076:01	public	4
DLA08348	DLA-88*079:01	public	4
DLA08349	DLA-88*511:02	public	4
DLA08323	DLA-88*002:02	public	3
DLA08345	DLA-88*030:02	public	3
DLA08342	DLA-88*049:03	public	3
DLA08327	DLA-88*060:03	public	3
DLA08338	DLA-88*075:01	public	3
DLA08341	DLA-88*077:01	public	3
MK617588.1	DLA-88*006m47	Not assigned	3
MK617585.1	DLA-88*N5m	Not assigned	3
MK617593.1	DLA-88*N7m1	Not assigned	2
MK617597.1	DLA-88*047:01m	Not assigned	2
MK617594.1	DLA-88*N2m036	Not assigned	2
MK617610.1	DLA-88*02801m2	Not assigned	2
MK617595.1	DLA-88*03401m	Not assigned	2
MW139898	DLA-88*novel25V	Not assigned	2
MK617592.1	DLA-88*N32m1	Not assigned	2
MK617613.1	DLA-88*03001m	Not assigned	1
MK617609.1	DLA-88*03901m	Not assigned	1
MN167163.1	DLA-88*042m40	Not assigned	1
MK617606.1	DLA-88*N31mN40	Not assigned	1

SUPPLEMENTAL TABLE 2. Complete DLA-88 allele list and frequencies found by NGS

Accession (IPD/GenBank)	Name	Status	Number of cases
MK617602.1	DLA-88*N45m49	Not assigned	1
MK617611.1	DLA-88*02501m	Not assigned	1
MK617612.1	DLA-88*00501m	Not assigned	1
MT543269.1	DLA-88*02601m2	Not assigned	1
MN188078.1	DLA-88*044m008	Not assigned	1
MK617590.1	DLA-88*N39m46	Not assigned	1
MK617607.1	DLA-88*AmN16	Not assigned	1
MK617579.1	DLA-88*N16m1	Not assigned	1
MT597426.1	DLA-88*N7m	Not assigned	1
MK617600.1	DLA-88*N46mN25	Not assigned	1
MK617603.1	DLA-88*N40m1	Not assigned	1
MK617598.1	DLA-88*002m04501	Not assigned	1
MN188080.1	DLA-88*Bm28	Not assigned	1
DLA08080	DLA-88*002:01	public	61
DLA08156	DLA-88*029:01	public	57
DLA08165	DLA-88*012:01	public	55
DLA08113	DLA-88*004:02	public	46
DLA07988	DLA-88*006:01	public	40
DLA08063	DLA-88*010:01	public	40
DLA08093	DLA-88*028:01	public	32
DLA08284	DLA-88*043:02	public	31
DLA08116	DLA-88*508:01	public	31
DLA07975	DLA-88*005:01	public	28
DLA08206	DLA-88*051:01	public	25
DLA08269	DLA-88*003:02	public	22
DLA08312	DLA-88*007:02	public	20
DLA08082	DLA-88*017:01	public	20
DLA08318	DLA-88*024:02	public	20
DLA08354	DLA-88*501:02	public	14
DLA08294	DLA-88*028:05	public	13
DLA08305	DLA-88*013:02	public	12
DLA08253	DLA-88*016:03	public	11
DLA08224	DLA-88*016:04	public	11
DLA08291	DLA-88*036:02	public	11
DLA08304	DLA-88*060:02	public	11
DLA08288	DLA-88*014:01:02	public	10
LC130518.1	DLA-88*novel25	Not assigned	9
DLA08086	DLA-88*022:01	public	9

SUPPLEMENTAL TABLE 2. Complete DLA-88 allele list and frequencies found by NGS

Accession (IPD/GenBank)	Name	Status	Number of cases
DLA08158	DLA-88*045:02	public	9
DLA08108	DLA-88*501:01	public	9
DLA08300	DLA-88*006:02	public	8
DLA08205	DLA-88*028:03	public	7
DLA08097	DLA-88*034:01	public	7
DLA08295	DLA-88*056:01	public	7
DLA08314	DLA-88*065:01	public	7
DLA08321	DLA-88*024:03	public	6
DLA08290	DLA-88*054:01	public	6
DLA08298	DLA-88*058:01	public	6
DLA08237	DLA-88*045:01	public	5
DLA08287	DLA-88*052:01	public	5
DLA08322	DLA-88*026:02	public	4
DLA08313	DLA-88*028:07	public	4
DLA08098	DLA-88*035:01	public	4
DLA08101	DLA-88*039:01:01	public	4
DLA08135	DLA-88*040:01	public	4
DLA08103	DLA-88*041:01	public	4
DLA08301	DLA-88*049:02	public	4
DLA08307	DLA-88*052:02	public	4
DLA08309	DLA-88*062:01	public	4
DLA08023	DLA-88*008:01	public	3
DLA08081	DLA-88*016:02	public	3
DLA08319	DLA-88*016:05	public	3
DLA08286	DLA-88*042:02	public	3
DLA08297	DLA-88*019:02	public	2
DLA08089	DLA-88*025:01	public	2
DLA08308	DLA-88*028:06	public	2
DLA08194	DLA-88*032:01	public	2
DLA08204	DLA-88*032:02	public	2
DLA08100	DLA-88*038:01	public	2
DLA08168	DLA-88*049:01	public	2
DLA08296	DLA-88*057:01	public	2
DLA08303	DLA-88*060:01	public	2
DLA08311	DLA-88*064:01	public	2
DLA08109	DLA-88*502:01	public	2
DLA08115	DLA-88*507:01	public	2
DLA08357	DLA-88*508:03	public	2

SUPPLEMENTAL TABLE 2. Complete DLA-88 allele list and frequencies found by NGS

Accession (IPD/GenBank)	Name	Status	Number of cases
DLA07967	DLA-88*001:01	public	1
DLA08299	DLA-88*003:03	public	1
DLA08005	DLA-88*007:01	public	1
DLA08078	DLA-88*015:01	public	1
DLA08278	DLA-88*021:01	public	1
DLA08320	DLA-88*029:02	public	1
DLA08198	DLA-88*036:01	public	1
DLA08292	DLA-88*043:04	public	1
DLA08169	DLA-88*046:01	public	1
DLA08306	DLA-88*061:01	public	1
DLA08310	DLA-88*063:01	public	1
DLA08261	DLA-88*001:03	public	0
DLA08091	DLA-88*003:01	public	0
DLA08102	DLA-88*004:01	public	0
DLA08042	DLA-88*009:01	public	0
DLA08331	DLA-88*010:02	pending	0
DLA08238	DLA-88*011:01	public	0
DLA08177	DLA-88*012:02	public	0
DLA08076	DLA-88*013:01	public	0
DLA08077	DLA-88*014:01:01	public	0
DLA08079	DLA-88*016:01	public	0
DLA08083	DLA-88*018:01	public	0
DLA08084	DLA-88*019:01	public	0
DLA08085	DLA-88*020:01	public	0
DLA08317	DLA-88*021:02	public	0
DLA08087	DLA-88*023:01	public	0
DLA08088	DLA-88*024:01	public	0
DLA08090	DLA-88*026:01	public	0
DLA08092	DLA-88*027:01	public	0
DLA08094	DLA-88*028:02	public	0
DLA08201	DLA-88*028:04	public	0
DLA08095	DLA-88*030:01	public	0
DLA08096	DLA-88*031:01	public	0
DLA08196	DLA-88*033:01	public	0
DLA08099	DLA-88*037:01	public	0
DLA08351	DLA-88*039:01:02	pending	0
DLA08104	DLA-88*042:01	public	0
DLA08105	DLA-88*043:01	public	0

SUPPLEMENTAL TABLE 2. Complete DLA-88 allele list and frequencies found by NGS

Accession (IPD/GenBank)	Name	Status	Number of cases
DLA08106	DLA-88*044:01	public	0
DLA08185	DLA-88*047:01	public	0
DLA08107	DLA-88*048:01	public	0
DLA08283	DLA-88*050:01	public	0
DLA08289	DLA-88*053:01	public	0
DLA08293	DLA-88*055:01	public	0
DLA08350	DLA-88*058:02	pending	0
DLA08302	DLA-88*059:01	public	0
DLA08315	DLA-88*066:01	pending	0
DLA08324	DLA-88*068:01	public	0
DLA08329	DLA-88*070:01	public	0
DLA08328	DLA-88*070:02	public	0
DLA08330	DLA-88*071:01	pending	0
DLA08335	DLA-88*074:01	abandoned	0
DLA08110	DLA-88*503:01	public	0
DLA08111	DLA-88*504:01	public	0
DLA08112	DLA-88*505:01	public	0
DLA08114	DLA-88*506:01	public	0
DLA08244	DLA-88*508:02	public	0
DLA08124	DLA-88*509:01	public	0
DLA08355	DLA-88*510:01	public	0
DLA08356	DLA-88*511:01	pending	0
DLA08398	DLA-88*512:02	pending	0
DLA08360	DLA-88*514:01	pending	0

Note: Green highlight signifies DLA-88L alleles, known to reside at the DLA-12 locus.[28]
Blue highlight signifies new alleles detected in this study.

SUPPLEMENTARY TABLE 3. New DLA-88 allele confirmation

Allele Name	Carrier dogs identified by NGS[a]	Carrier dogs confirmed by Sanger[b]	Carrier dogs missed by Sanger[c]
DLA-88*072:01	4	4	0
DLA-88*067:01	3	3	0
DLA-88*050:03	4	3	1
DLA-88*067:02	3	3	0
DLA-88*01601N25m	3	3	0
DLA-88*046:02	4	2	2
DLA-88*069:01	4	2	2
DLA-88*078:01	3	3	0
DLA-88*069:02	5	5	0
DLA-88*512:01	3	3	0
DLA-88*079:01	4	2	2
DLA-88*073:01	3	3	0
DLA-88*050:02	3	3	0
DLA-88*513:01	3	1	2
DLA-88*043:03	3	3	0
DLA-88*030:02	1	1	0
DLA-88*075:01	3	3	0
DLA-88*076:01	3	3	0
DLA-88*077:01	3	3	0
DLA-88*060:03	3	3	0
DLA-88*049:03	3	3	0
DLA-88*N5m	3	3	0
DLA-88*008:02	3	2	1
DLA-88*006m47	3	0	3
DLA-88*002:02	3	3	0
DLA-88*N32m1	2	1	1
DLA-88*N7m1	2	1	1
DLA-88*N2m036	2	2	0
DLA-88*03401m	2	2	0
DLA-88*047:01m	2	2	0
DLA-88*N16m1	1	1	0
DLA-88*N39m46	1	1	0
DLA-88*002m04501	1	1	0
DLA-88*042m40	1	0	1
DLA-88*AmN16	1	0	1
DLA-88*Bm28	1	0	1

[a]Number of dogs identified as carriers of the allele among the 63 dogs tested.
[b]Number of carriers confirmed by Sanger method out of 63 dogs re-tested.
[c]Number of NGS calls missed by Sanger method out of 63 dogs re-tested.

SUPPLEMENTAL TABLE 4. Complete DLA88 allele list and frequencies found in two breeds

Great Dane (n=48)		
**Allele**	**Number**	**(%)**
DLA-88*012:01	35	33.0
DLA-88*043:02	23	21.7
DLA-88*051:01	14	13.2
DLA-88*004:03	8	7.5
DLA-88*010:01	6	5.7
DLA-88*016:03	3	2.8
DLA-88*016:02	3	2.8
DLA-88*040:01	3	2.8
DLA-88*006:01	2	1.9
DLA-88*novel25V	2	1.9
DLA-88*004:02	1	0.9
DLA-88*005:01	1	0.9
DLA-88*022:01	1	0.9
DLA-88*069:02	1	0.9
DLA-88*069:01	1	0.9
DLA-88*006:02	1	0.9
DLA-88*013:02	1	0.9

German Shepherd (n=48)		
**Allele**	**Number**	**(%)**
DLA-88*002:01	44	36.1
DLA-88*004:02	20	16.4
DLA-88*010:01	19	15.6
DLA-88*006:01	9	7.4
DLA-88*045:02	7	5.7
DLA-88*003:02	4	3.3
DLA-88*017:01	4	3.3
DLA-88*022:01	3	2.5
DLA-88*005:01	2	1.6
DLA-88*029:01	2	1.6
DLA-88*016:04	2	1.6
DLA-88*novel25	2	1.6
DLA-88*002m04501	1	0.8
DLA-88*038:01	1	0.8
DLA-88*508:01	1	0.8
DLA-88*501:02	1	0.8

Note: Green highlight signifies DLA-88L alleles, known to reside at the DLA-12 locus.[28]

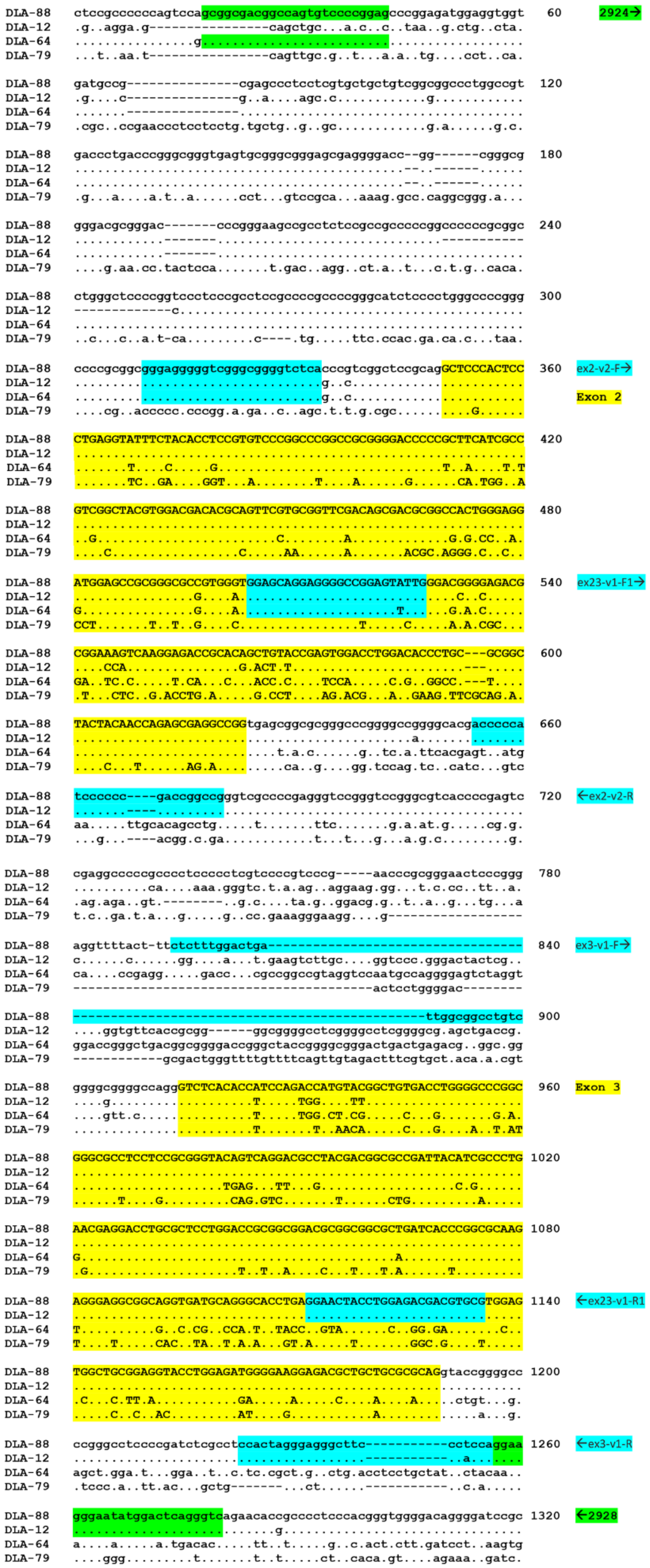

SUPPLEMENTAL FIGURE 1. Positions of PCR primers on DLA Class 1 Genes: The polymorphic region of DLA-88, (exons 2 and 3) along with flanking sequence, is aligned with the other 3 well-documented Class 1 DLA genes, DLA-12, DLA-64 and DLA-79. Sequences were taken from the canine genomic data base (CanFam3.1) on the UCSC genome browser (https://genome.ucsc.edu/). Base position numbers are arbitrary, relating to the first base of the aligned sequence. Exons 2 and 3 are highlighted yellow. Dots indicate identity to DLA88. Dashes indicate gaps in alignment. Positions of the 3 sets of PCR primers used for amplifying the exon 2, exon 3 and bridge regions for NGS sequencing of DLA-88 are highlighted in blue. Homologous positions on the other class 1 genes, that are potential targets, are also highlighted. Based on the sequences shown, the exon 2 and bridge primer pairs could potentially amplify alleles of both DLA-88 and DLA-12, while the exon 3 primer pair should be specific to DLA-88. However, variations in these genes, that could alter the specificity of the primers, are known to occur throughout this region. Primers used for the Sanger sequencing method are highlighted in green. This primer pair is specific to DLA-88.

A Carriers of 3 or more alleles[a]

Total dogs in study	Carrier Dogs
382	176

B DLA-88 carriers

DLA-88*016:03	DLA-88*016:04	DLA-88*016:05	DLA-88*017:01	DLA-88*024:02	DLA-88*024:03	DLA-88*029:01	DLA-88*029:02	DLA-88*026:02	Total dogs in study	Total Carrier Dogs
11	11	3	20	20	6	57	1	4	382	133[b]

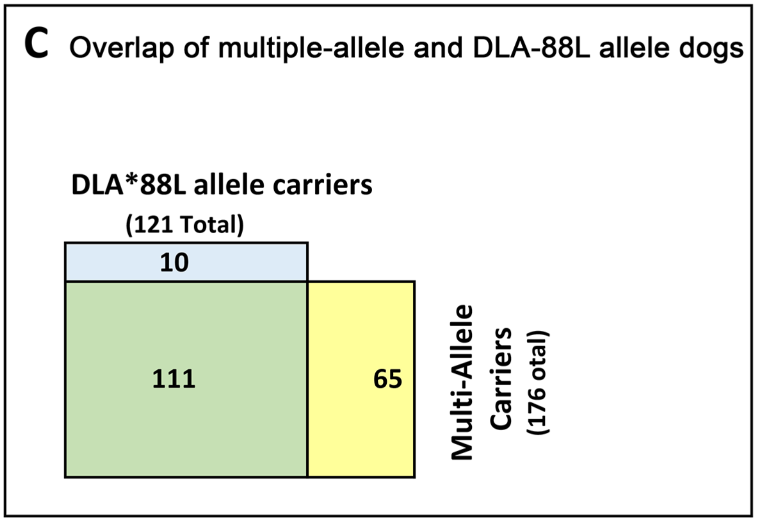

SUPPLEMENTAL FIGURE S2. Distribution of "extra" DLA-88 alleles among dogs in this study.

[a] Multiple-allele dogs are those dogs for which more than 2 DLA-88 alleles were detected by NGS.

[b] Note that some DLA-88L carriers have more than one DLA-88L allele.
